# Contrast-enhanced ultrasound tractography for 3D vascular imaging of the prostate

**DOI:** 10.1038/s41598-018-32982-2

**Published:** 2018-10-02

**Authors:** Ruud J. G. van Sloun, Libertario Demi, Stefan G. Schalk, Cristina Caresio, Christophe Mannaerts, Arnoud W. Postema, Filippo Molinari, Hans C. van der Linden, Pingtong Huang, Hessel Wijkstra, Massimo Mischi

**Affiliations:** 1 Department of Electrical Engineering, Eindhoven University of Technology, Eindhoven, The Netherlands; 2 Department of Urology, Academic Medical Center, University of Amsterdam, Amsterdam, The Netherlands; 3 Department of Electronics and Telecommunications, Biolab, Polytechnic University of Turin, Turin, Italy; 4 Department of Pathology/DNA laboratories, Jeroen Bosch Hospital, ‘s-Hertogenbosch, The Netherlands; 5 Department of Ultrasound, Second Affiliated University Hospital, Zhejiang University School of Medicine, Hangzhou, PR China; 6 Department of Information Engineering and Computer Science, University of Trento, Trento, Italy

## Abstract

Diffusion tensor tractography (DTT) enables visualization of fiber trajectories in soft tissue using magnetic resonance imaging. DTT exploits the anisotropic nature of water diffusion in fibrous structures to identify diffusion pathways by generating streamlines based on the principal diffusion vector. Anomalies in these pathways can be linked to neural deficits. In a different field, contrast-enhanced ultrasound is used to assess anomalies in blood flow with the aim of locating cancer-induced angiogenesis. Like water diffusion, blood flow and transport of contrast agents also shows a principal direction; however, this is now determined by the local vasculature. Here we show how the tractographic techniques developed for magnetic resonance imaging DTT can be translated to contrast-enhanced ultrasound, by first estimating contrast flow velocity fields from contrast-enhanced ultrasound acquisitions, and then applying tractography. We performed 4D *in-vivo* contrast-enhanced ultrasound of three human prostates, proving the feasibility of the proposed approach with clinically acquired datasets. By comparing the results to histopathology after prostate resection, we observed qualitative agreement between the contrast flow tracts and typical markers of cancer angiogenic microvasculature: higher densities and tortuous geometries in tumor areas. The method can be used *in-vivo* using a standard contrast-enhanced ultrasound protocol, opening up new possibilities in the area of vascular characterization for cancer diagnostics.

## Introduction

The introduction of diffusion tensor tractography (DTT) in magnetic resonance imaging (MRI) led to numerous new studies in brain research^1^. This was the first non-invasive *in-vivo* imaging modality enabling the generation of fiber trajectories in soft fibrous tissues, such as nerves and muscles^2^. Relevant information about neural network connectivity, white matter deficits and tumor infiltration are now all available through DTT. The method has widespread potential implications in both cognitive neuroscience and neurobiology^3,4^. At the core of DTT lies the anisotropic nature of water diffusion in white matter. Water molecules have a preferred direction of diffusion in fibrous structures, a directionality that can be tracked and displayed with streamlines^5^. As such, DTT became a widely used technique to visualize bundles of nerves in the brain. In this work, we translate these tractographic technologies to contrast-enhanced ultrasound (CEUS), which has now advanced to a level that permits detection of contrast-agent concentrations in 4D (3D space + time)^6^. Where DTT MRI is used to visualize white matter axons, CEUS tractography will be employed to visualize vascular patterns, thereby enabling assessment of typical angiogenic features in cancer such as higher vascular densities and tortuous geometries^7^.

In CEUS, the passage of an intravenously injected bolus of ultrasound contrast agent through an organ is recorded with an ultrasound imaging system^8^. These lipid-shelled microbubbles have a size similar to red blood cells and therefore remain intravascular while reaching the smallest capillaries in the vascular net. CEUS has been adopted in clinical practice with applications ranging from cardiology to oncology^9^. For the latter, clinicians mainly rely on qualitative inspection of the ultrasound videos^10^, searching for visual clues such as early contrast enhancement. Although recent developments in quantitative interpretation of CEUS videos have shown promise^11–13^, a technique that provides explicit information on the underlying vascular architecture using standard clinical CEUS protocols is still hampered by the resolution limits of clinical ultrasound scanners.

Current methods for direct *in-vivo* vascular imaging using CEUS are indeed challenging to fit into clinical protocols. If one extends beyond the regular protocols and permits longer acquisition times, a recently proposed technique named ultrafast ultrasound localization microscopy can be considered^14^. This approach visualizes the microvascular architecture with astonishing resolution by borrowing concepts from photo-activated localization microscopy^15^. However, super-localization technologies remain highly sensitive to motion artifacts, complicating their clinical application. Moreover, their 3D implementation is currently restricted by data overload as well as challenging probe design and electronics^16^. Another ultrasound-based approach for microvascular imaging is acoustic angiography^17^. It embraces the high frequency content generated by resonating microbubbles to reach resolutions that can resolve vessels with diameter down to 150 µm. While these high frequencies are an asset for achieving a high spatial resolution, they are also the method’s main drawback: Penetration depth is limited as acoustic absorption by tissue strongly increases with frequency. Moreover, its peculiar imaging strategy requires the use of very broad-banded ultrasound transducers (roughly 2 to 45 MHz) or specially designed dual-frequency probes.

Here, we propose contrast-enhanced ultrasound tractography (CEUS-T), which combines revolutionary concepts from DTT MRI for fiber visualization with CEUS blood flow vector imaging^13^, yielding 3D images of contrast agent trajectories. The analogy with DTT MRI is clear: similar to diffusion of water molecules in fibers, microbubbles move through an organ with a directionality that is now determined by the vascular architecture. CEUS-T was performed *in-vivo* using 4D CEUS recordings of three human prostates obtained in a clinical setting, with a clinically approved ultrasound system and ultrasound contrast agents.

## Results

### Imaging principle flow orientations on low volume rate 4D CEUS

**Figure 1:**
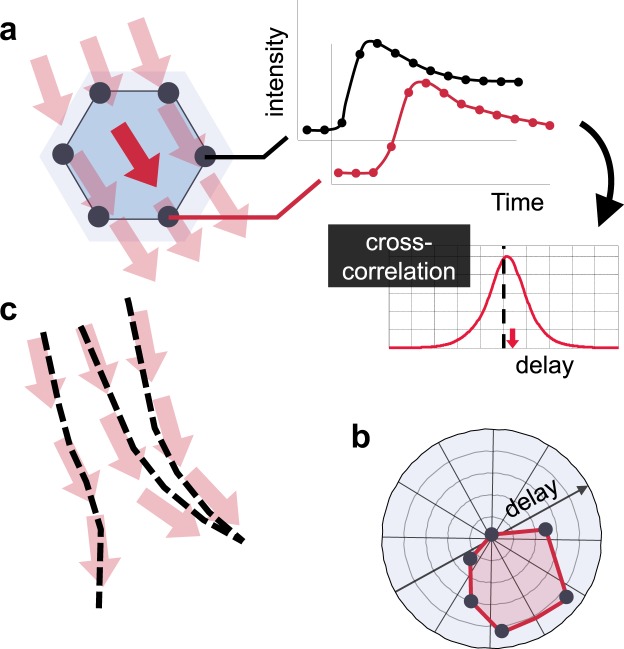
Principle of CEUS-T (**a**) Time-delay estimation amongst a set of time-intensity curves. (**b**) Time-delay magnitude as a function of orientation. (**c**) Velocity vector estimation and tractography.

At the core of CEUS-T lies the estimation of flow vector fields from 4D CEUS. The low volume rates obtained with standard 4D CEUS, typically even below one volume per second, pose a major problem for standard velocity vector imaging based on speckle tracking or optical flow^18^. Severe spatial de-correlation occurs between subsequent frames. While the temporal resolution is hence not sufficient to track microbubbles over space, it is sufficiently high to capture the average kinetics of a diffused cloud of microbubbles^6^. As such, all the required phase information to determine the bolus transit time from one point in space to another point in space is retained. The usefulness of this property becomes evident when comparing the transit times, or time-delays, amongst contrast time-intensity curves obtained at a spatially distributed set of voxels surrounding the location of interest. The relation between this specific set of curves can be visualized in a delay-orientation distribution function^19^ (Fig. 1a,b), reflecting the dominant flow directivity.

To achieve an accurate delay estimate at a sufficiently high temporal resolution, we interpolate all time signals by a factor 10 using Sinc-interpolation^20^. As the dominant bandwidth of the contrast agent evolution over time is mostly below the Nyquist frequency in the prostate^6^, this approach allows near-perfect reconstruction of the true signal values at the interpolated sample-points. Using these interpolated dynamic CEUS datasets, the dominant microbubble flow velocity vector at each voxel can then be obtained by constructing the aforementioned delay-orientation distribution function based on the delays from time-intensity curves measured at surrounding voxels.

We estimate these time-delays amongst the 113 antipodal pairs at over 6 million locations in the imaging volume, accounting for more than 600 million evaluations of this procedure. For each of those 6 million voxels in the 3D volume, the dominant flow orientation and magnitude is then obtained by minimizing the squared error between a model-prediction of the time-delays given a velocity vector, and the measured time-delays^13^. This facilitates the estimation of a 3D velocity field of microbubble propagation through the organ.

### CEUS tractography

After generating the full 3D representation of propagation directions, we exploit tractography^21^ to visualize flow trajectories. As for DTT, this translates into solving the differential equation that describes the movement of a particle within the estimated vector fields, given an initial seed point (Fig. 1c). A complete CEUS-T image is formed by rendering the trajectories originating from many of these points distributed uniformly across the imaging space. We render the trajectories as semi-transparent color-coded lines, emphasizing those paths that are followed by many streamlines as opposed to the ones that originate from only few. The color coding can for instance reflect the macroscopic velocity magnitude (as calculated from the local time-delay distributions) or a tract feature (e.g. tortuosity).

**Figure 2:**
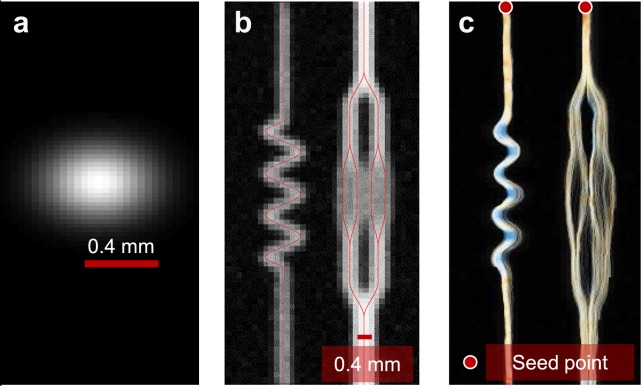
2D CEUS-T on synthetic data (**a**). The adopted imaging point spread function. Maximum echo intensity for a simulation of contrast agent transport through an artificial branching structure (**b**). Multiple streamlines originating from indicated seed points (**c**).

Figure 2 demonstrates the ability of CEUS-T to identify flow trajectories from this synthetic *in-silico* data of microbubble transport through a branching network. Multiple streamlines are generated from the indicated seed-points based on a probabilistic approach, displaying the four branches of the vascular tree that are not visible in the standard maximum ultrasound intensity image.

**Figure 3:**
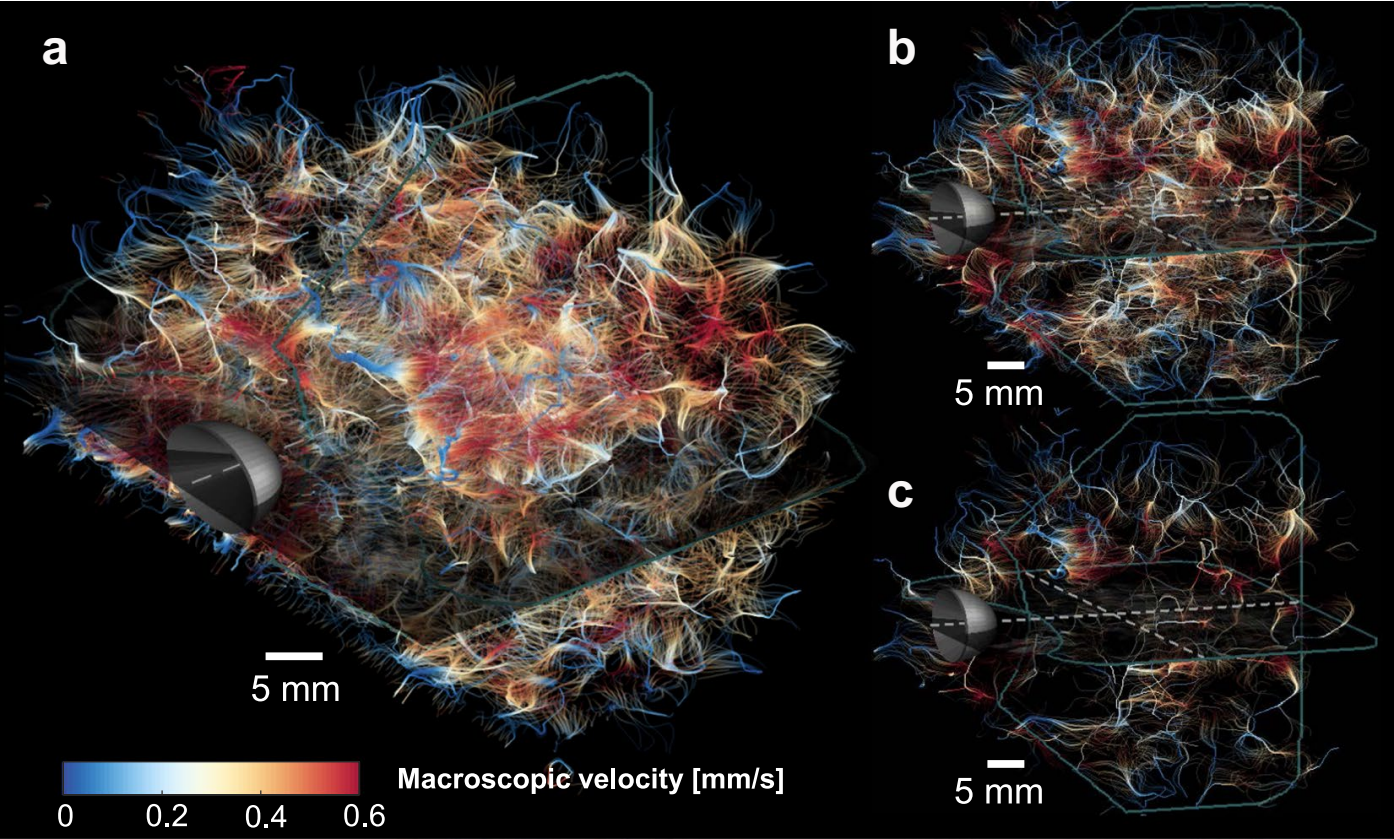
3D CEUS-T of a human prostate. (**a**) CEUS-T image, displaying the network of trajectories obtained by applying the method to a transrectally recorded CEUS sequence of a human prostate. (**b**) CEUS-T image where only trajectories longer than 5 mm are displayed. (**c**) Only trajectories longer than 8 mm are displayed. Colors depict the macroscopic flow velocity.

When applying CEUS-T to an *in-vivo* 4D CEUS recording of a human prostate, we obtain a dense network of trajectories, with pathways of high incidence revealing what are likely vascular structures (Fig. 3). We can regulate the density of streamlines by visualizing only those trajectories that have a path length above a certain threshold, as shown in Fig. 3b,c. The longest trajectories were about 25 mm.

### CEUS tractography compared with histopathology

**Figure 4:**
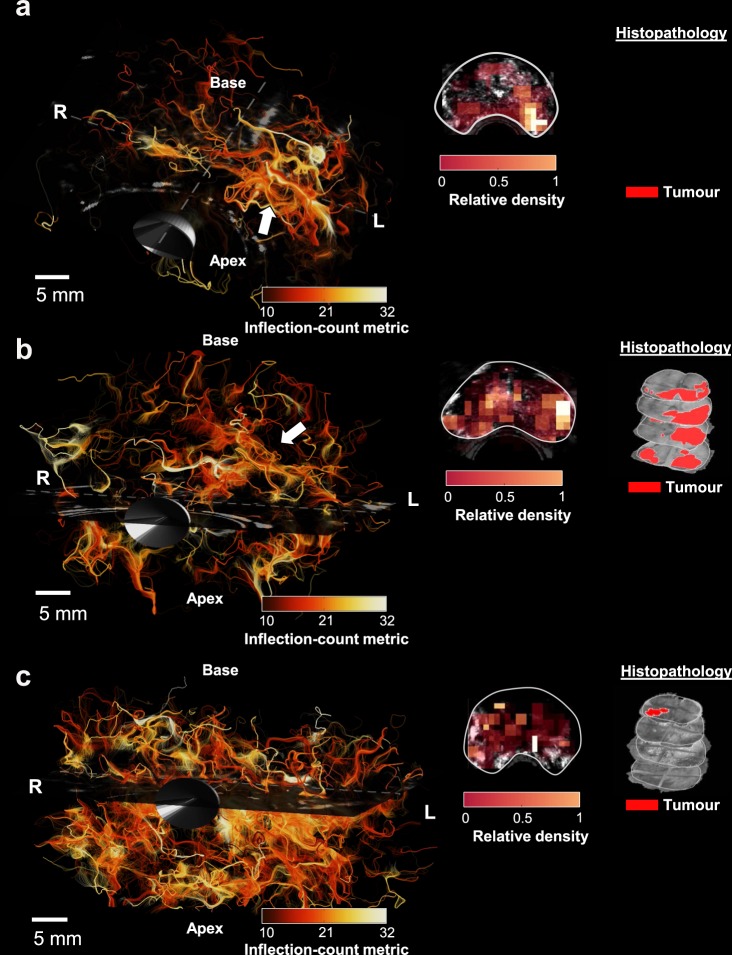
3D CEUS-T with tortuosity quantification and density maps on 3 different human prostates with histology. Colors encode the tract inflection count metric (ICM). (**a**) Prostate I: CEUS-T displays elevated ICM and higher tract density on the left mid-base side of the prostate. Histology reveals a malignant lesion (Gleason score: 4 + 5 = 9), with a left mid- basal focus. (**b**) Prostate II: CEUS-T yields a higher tract density on the complete left side of the prostate. Histology reveals a malignant lesion on the left (Gleason score: 5 + 5 = 10) and another significant lesion with a right apical focus (Gleason score: 4 + 5 = 9). (**c**) Prostate III: CEUS-T shows a generally dense image. Here histology yielded benign prostate hyperplasia across the entire gland and a small malignant lesion (Gleason score: 3 + 3 = 6) in the basis.

More *in-vivo* prostate examples are given in Fig. 4. These cases come from patients that were diagnosed with prostate cancer, and referred for radical prostatectomy. Here, only trajectories that have a minimum Euclidian distance of 10 mm between the start- and end-points are displayed. We encoded CEUS-T tracts with colors that represent a tract-feature related to tortuosity: the inflection-count metric (ICM). In addition, 2D density maps were derived by counting the number of trajectories passing through sub-volumes of 2.5 × 2.5 × 5 mm at a mid-basal slice. In case one (4a), the image displays a denser network of trajectories on the left side of the prostate, with elevated ICM values. A corresponding malignant lesion (Gleason score 4 + 5 = 9) was found by histology. A similar CEUS-T pattern can be observed for case two (4b), where histology revealed a malignant lesion (Gleason score 5 + 5 = 10) on the complete left side of the prostate, and another lesion with a right apical focus (Gleason score: 4 + 5 = 9). In case three, the CEUS-T image generally appears very dense. Interestingly, vast benign prostate hyperplasia (BPH) was found across the entire gland along with a small low-grade lesion (Gleason grade: 3 + 3 = 6). Like prostate carcinoma, BPH is also associated with angiogenesis, even if to a lesser extent^22,23^.

## Discussion

We demonstrated that the techniques developed for DTT magnetic resonance imaging can be applied to 3D microbubble flow vector fields obtained from CEUS to attain CEUS-T images. CEUS-T has the remarkable ability to visualize 3D microbubble flow trajectories in a comprehensible and effective manner. The method can be applied effectively to standard CEUS data that is acquired with a clinical ultrasound scanner in a clinical setting using approved contrast agents.

In two out of three prostate-cancer cases, the spatial density of trajectories qualitatively showed an excellent agreement with the malignancies found by histopathology. Interestingly, the third case displayed widespread benign hyperplasia, also associated with the presence of angiogenesis. These results suggest that the information captured by CEUS-T can be used to assess vascular characteristics associated to angiogenesis.

This study aimed at providing a proof-of-principle, including a qualitative evaluation on three cancer cases. As such, no quantitative assessment was performed to avoid drawing quantitative conclusions based on three samples only, which cannot be representative for the heterogeneous prostate-cancer population. To assess the method’s diagnostic and clinical implications, extensive validation on a larger group of patients should be carried out.

The proposed qualitative validation was performed through comparison with histological assessment of the Gleason score from H&E stained prostate specimens, which is based on degree of cell differentiation. While this provides a good indication of malignancy and aggressiveness, it does not describe explicit vascular characteristics. The absence of a vascular ground truth prevents *in-vivo* assessment of the absolute correctness of the rendered vessel trajectories. Notwithstanding the usefulness of such an assessment, it is in practice indeed extremely challenging to obtain such a ground truth.

Although the fiber tracking methods behind magnetic resonance DTT served as an inspiration for the method presented in this paper, directional diffusion of water in fiber bundles is very distinct from the motion of microbubbles in vessels, and the spatial resolutions of the two techniques are rather different. Nevertheless, these concepts differentiate CEUS-T from traditional techniques such as maximum-intensity-projection (MIP) imaging, opening up towards the creation of new color maps that can provide a valuable contribution to clinical diagnostics. One can for instance map various image-derived features such as flow velocity or trajectory tortuosity and density.

CEUS-T captures aspects from all scales of the vasculature, but its ability to resolve those individual vessels is influenced by the size of the kernel used to estimate the velocity vectors, which is in turn limited by the resolution of the ultrasound system. Careful assessment of the *in-silico* results (Fig. 2) reveals that imaging at the diffraction limit itself lies at the edge of the method’s abilities; while the final bifurcation on left main branch is resolved nicely, the other side displays a more dispersed collection of tracts. This phenomena also occurs *in-vivo*, where compact bundles of trajectories ultimately branch into a diffused cloud of tracts at the capillary level. Another limiting factor lies in the precision of the time-delay estimates, which is in turn influenced by the accuracy of the temporal interpolation. Although the contrast-agent bolus dynamics are below the temporal Nyquist frequency of the imaging system, perfect signal recovery is only guaranteed in the absence of broadband noise. The latter would give rise to colored noise after Sinc-interpolation of the sampled signals, making the adopted cross-correlation time-delay estimator deviate from the maximum likelihood estimator. The impact of this depends on the degree of coloration, and therewith on the frame rate of the system. Given these considerations, the fidelity of CEUS-T will likely improve with the resolution and frame rate of the imaging system.

Currently, CEUS-T velocity vector estimates are directly obtained from the time-delay distribution within a kernel by calculating an average propagation vector. This poses a limitation for pairs of arterioles and venules that fall within one kernel and run side-by-side with flow the opposite direction. In future work, one could envisage solving the velocity estimation problem as a ‘compressed sensing’ inverse problem, in which multiple sparse underlying propagation directions are possible within one kernel. Similar extended algorithms have been proposed in magnetic resonance DTT for resolving multiple crossing fibers within a resolution cell^24^. Another extension inspired by research in DTT is the use of probabilistic tractography, in which posterior density functions of trajectories (sampled in a Monte Carlo fashion) are rendered rather than deterministic tracts^25^. This enables quantification of uncertainty.

While the full potential and diagnostic value of CEUS-T remains to be investigated, it is conceivable that it will provide a relevant asset in many clinical applications where imaging vascular characteristics is of interest. The cases shown in this study are related to the detection of prostatic malignancies; yet CEUS-T is in principle suited to any dynamic CEUS recording of any organ. As such, it carries a widespread potential.

## Materials and Methods

### 2D in-silico data generation

The 2D transport of ultrasound contrast agents through a synthetic branching structure (laminar, plug flow) was simulated by propagating 2000 particles through the structure with a given velocity, comprising a deterministic flow (magnitude 1 mm/s) plus a Gaussian process noise component (zero mean with standard deviation 0.5 mm/s). The former simulates pure convection, whereas the latter served as a model for diffusion. The ultrasound acquisition was simulated by modelling the scanner’s point spread function as a bivariate Gaussian, with standard deviations that reflect the axial (*σ*_*x*_ = 0.14 mm) and lateral (*σ*_*y*_ = 0.16 mm) resolution. The frame rate was set to 10 Hz, and the pixel spacing was 0.15 mm.

### 4D CEUS *in-vivo* data acquisition

The 4D transrectal CEUS acquisitions were performed at the Second Affiliated Hospital of the Zhejiang University School of Medicine (Hangzhou, Zhejiang, China). Using an intravenous cannula in the arm, patients received a bolus injection of 2.4-ml SonoVue® followed by a 5 ml bolus injection of Saline, whose passage through the prostate was imaged using a 3D transrectal ultrasound probe (RIC5-9) and a LOGIQ E9 ultrasound scanner (GE Healthcare, Wauwatosa, WI, USA). A contrast-specific imaging mode was employed, and the organ was imaged for 2 minutes. The imaging quality setting was set to “low” in order to reach a volume rate of approximately 0.3 Hz. The voxel size is 0.25 × 0.25 × 0.25 mm. The *in-vivo* studies received prior approval from the institutional review board of the Second Affiliated Hospital of Zhejang University, and were performed in accordance with the guidelines and regulations. Informed consent was obtained from all patients. The 3D datasets were up-sampled by a factor 10 prior to the analysis using Whittaker-Shannon interpolation by zero-padding the temporal fast Fourier transform (FFT) of the input dataset, after which an inverse FFT was performed^20^.

### Velocity vector estimation

A local estimation of the microbubble flow velocity and directionality is obtained by considering the time-intensity relation amongst specific set of data points: N = 226 voxels spatially distributed on a sphere with a radius of 0.8 mm around the origin, accounting for the resolution of the employed imaging system. The antipodes (pairs of voxels that are mirrored with respect to the origin) are selected, and the time delays amongst those time-intensity curves are then estimated by determining the peak of their cross-correlation functions^13^. The local propagation velocity vector ***v*** = [*v*_*x*_, *v*_*y*_, *v*_*z*_] can then be estimated by solving the following linear system of equations using least-squares:

1 $${{\boldsymbol{v}}}^{T}{\boldsymbol{\tau }}=D,$$

Where ***τ*** is the row vector that contains all the estimated time delays and D is the matrix that describes the inter-voxel distance vectors. This procedure is repeated for all pixels in order to produce a velocity vector field.

### Anisotropic filtering

The obtained vector fields are smoothed using an anisotropic Gaussian filter that promotes velocity vectors that share a similar orientation with neighboring voxels^26^. The standard deviation of this oriented filter is 2 voxels in the direction of ultrasound-contrast-agent propagation, whereas this value is set to 1 voxel in the directions orthogonal to this component.

### Tractography

The streamlines of the velocity field are obtained by solving the following ordinary differential equation^27^:

2 $$\{\begin{array}{ccc}{{\boldsymbol{\partial }}}_{{\boldsymbol{t}}}{\boldsymbol{x}}(t) & = & {\boldsymbol{v}}[{\boldsymbol{x}}(t)]\\ {\boldsymbol{x}}(0) & = & {{\boldsymbol{x}}}_{0}\end{array},$$

which describes how, given an initial seeding point ***x***_0_, a particle moves within the velocity vector field ***v***[***x***]. We employ an explicit Euler method to solve (2), as implemented in the MATLAB (MathWorks, Massachusetts, United States) stream3c function. The seeding points of the algorithm are distributed uniformly across the imaging space, sub-sampled by a factor 3 in each direction; this amounts to roughly one point per resolution cell. Selecting too few seed points can not only lead to reduced spatial coverage, but also to artifacts in which isolated tracts within a laminar flow field of a single large vessel appear as several individual vessels. The maximum number of streamline vertices is set to 1000 and the integration step size is 0.1 (one-tenth of a cell). We finally re-sample the resulting tracts based on a nearest-neighbor scheme such that the distance between two samples is approximately 0.1 mm.

### Tortuosity

Tortuosity is assessed by calculating the inflection-count metric (ICM) of each tract^28^. The ICM calculates the number of inflection points along the tract and multiplies this number (plus 1) by the total path length of the curve divided by the distance between endpoints. We note that tortuosity metrics are susceptible to artifacts, underlining the importance of adequate anisotropic filtering prior to tractography.

### Histopathological analysis

After surgical resection, the prostate glands were fixed in formalin and the prostate was dissected into slices of 4-mm thickness. The slices were Haematoxylin/Eosin stained, and a pathologist determined the presence and extend of the tumour based on the degree of cell differentiation, according to the International Society of Urological Pathology (ISUP) consensus recommendations^29^.
